# Epidemiological characteristics and climatic variability of viral meningitis in Kazakhstan, 2014–2019

**DOI:** 10.3389/fpubh.2022.1041135

**Published:** 2023-01-04

**Authors:** Sauran Yerdessov, Assel Zhunussova, Aliya Imanova, Arnur Gusmanov, Yesbolat Sakko, Gulnur Zhakhina, Kamilla Mussina, Dmitriy Syssoyev, Aidar Alimbayev, Anara Abbay, Antonio Sarria-Santamera, Abduzhappar Gaipov

**Affiliations:** ^1^Department of Science and Education, CF “University Medical Center”, Astana, Kazakhstan; ^2^Department of Medicine, Nazarbayev University School of Medicine, Astana, Kazakhstan; ^3^Stroke Center, City Multidisciplinary Hospital No. 2, Astana, Kazakhstan; ^4^Clinical Academic Department of Internal Medicine, CF “University Medical Center”, Astana, Kazakhstan

**Keywords:** epidemiology, viral meningitis, in-hospital mortality, infectious diseases, seasonality, Kazakhstan

## Abstract

**Background:**

The comprehensive epidemiology and impact of climate on viral meningitis (VM) in Kazakhstan are unknown. We aimed to study the incidence, in-hospital mortality and influence of climatic indicators on VM from 2014 to 2019.

**Methods:**

Nationwide electronic healthcare records were used to explore this study. ICD-10 codes of VM, demographics, and hospital outcomes were evaluated using descriptive statistics and survival analysis.

**Results:**

During the 2014–2019 period, 10,251 patients with VM were admitted to the hospital. 51.35% of them were children, 57.85% were males, and 85.9% were from the urban population. Enteroviral meningitis was the main cause of VM in children. The incidence rate was 13 and 18 cases per 100,000 population in 2014 and 2019, respectively. Case fatality rate was higher in 2015 (2.3%) and 2017 (2.0%). The regression model showed 1°C increment in the daily average temperature might be associated with a 1.05-fold (95% CI 1.047–1.051) increase in the daily rate of VM cases, 1hPa increment in the average air pressure and 1% increment in the daily average humidity might contribute to a decrease in the daily rate of VM cases with IRRs of 0.997 (95% CI 0.995–0.998) and 0.982 (95% CI 0.981–0.983), respectively. In-hospital mortality was 35% higher in males compared to females. Patients residing in rural locations had a 2-fold higher risk of in-hospital death, compared to city residents. Elderly patients had a 14-fold higher risk of in-hospital mortality, compared to younger patients.

**Conclusion:**

This is the first study in Kazakhstan investigating the epidemiology and impact of climate on VM using nationwide healthcare data. There was a tendency to decrease the incidence with outbreaks every 5 years, and mortality rates were higher for Russians and other ethnicities compared to Kazakhs, for males compared to females, for elder patients compared to younger patients, and for patients living in rural areas compared to city residents. The climatic parameters and the days of delay indicated a moderate interaction with the VM cases.

## 1. Introduction

Meningitis is one of the global health problems affecting the central nervous system (CNS). According to WHO the disease caused about 250,000 deaths in 2019, leaving one in five affected individuals with long-term health sequelae and financial burden (1). Meningitis is defined as an inflammation of the meninges, the layers covering the brain and spinal cord, caused by a variety of factors, most frequently by microbial agents such as bacteria and viruses.

VM which is also termed aseptic meningitis is more common than bacterial meningitis (2). Typically, VM is a self-limited disease with a benign clinical course and better outcome than bacterial meningitis (3). However, several studies suggest that VM may lead to morbidity including neurocognitive and sleep disorders, and even mortality in patients, particularly in children (4–7). Various viral agents can cause meningitis: the most reported ones are enteroviruses and herpesviruses, but their relative incidence differs by country. Enteroviruses are responsible for the majority of VM cases in Spain, England, and the United States, whereas in Finland, for example, herpesviruses predominate, particularly *Human herpesvirus 2* and *Varicella-zoster virus* (3, 8–10).

Enteroviral meningitis appears to have a strong seasonal pattern. In regions with a temperate continental climate, enteroviral disease incidence generally increases during summer and early autumn. The mechanisms underlying this observation are not well-known, however, there are some proposed theories. Green et al. suggest that during summer and autumn, there is a potentially increased fecal-oral transmission of enteroviruses aided by warm weather and sparse clothing, especially among children (10).

Kazakhstan is the largest Central Asian country, with a wide spectrum of topographic variations and climate, ranging from a temperate climate in the north to a warm continental climate in the southern regions. Different landscapes and atmospheric conditions support a variety of fauna and consequently diverse endemicity of infectious agents in various regions of the country. For example, tick-borne illnesses, including encephalitis are well-reported in southern regions and are under constant vigilance (11, 12). But the national epidemiological situation of meningitis in Kazakhstan is unknown. With the exception of a surveillance study for meningitis and encephalitis in a high-endemic area of South Kazakhstan (13), there were no studies conducted related to VM both in adults and children at a country level. Generally, epidemiological studies are critical in understanding disease trends and relationships, which may contribute to disease control (7). With the intent of filling that gap, we conducted a nationwide epidemiological study looking into VM in the country.

The primary goal of this population-based study is to evaluate data from the unified nationwide electronic healthcare system to depict the epidemiologic profile, including incidence, and in-hospital mortality rates among the Kazakhstani population over 6 years. The secondary goal is to investigate the possible relationship of VM with common climatic variables in the country. We expect our study will improve knowledge of disease epidemiology in the country leading to improving evidence-based decisions in prevention, diagnostics, and treatment toward better managing patients with VM.

## 2. Materials and methods

### 2.1. Study population and data sources

This is a retrospective study of the Kazakhstani population hospitalized with VM according to the International Statistical Classification of Diseases and Related Health Problems (ICD-10) from 2014 to 2019. The following ICD-10 codes were utilized to identify patients with VM: A87.0, A87.2, A87.8, A87.9, B26.1, G02.0, G03.0, and G03.9.

**Figure 1:**
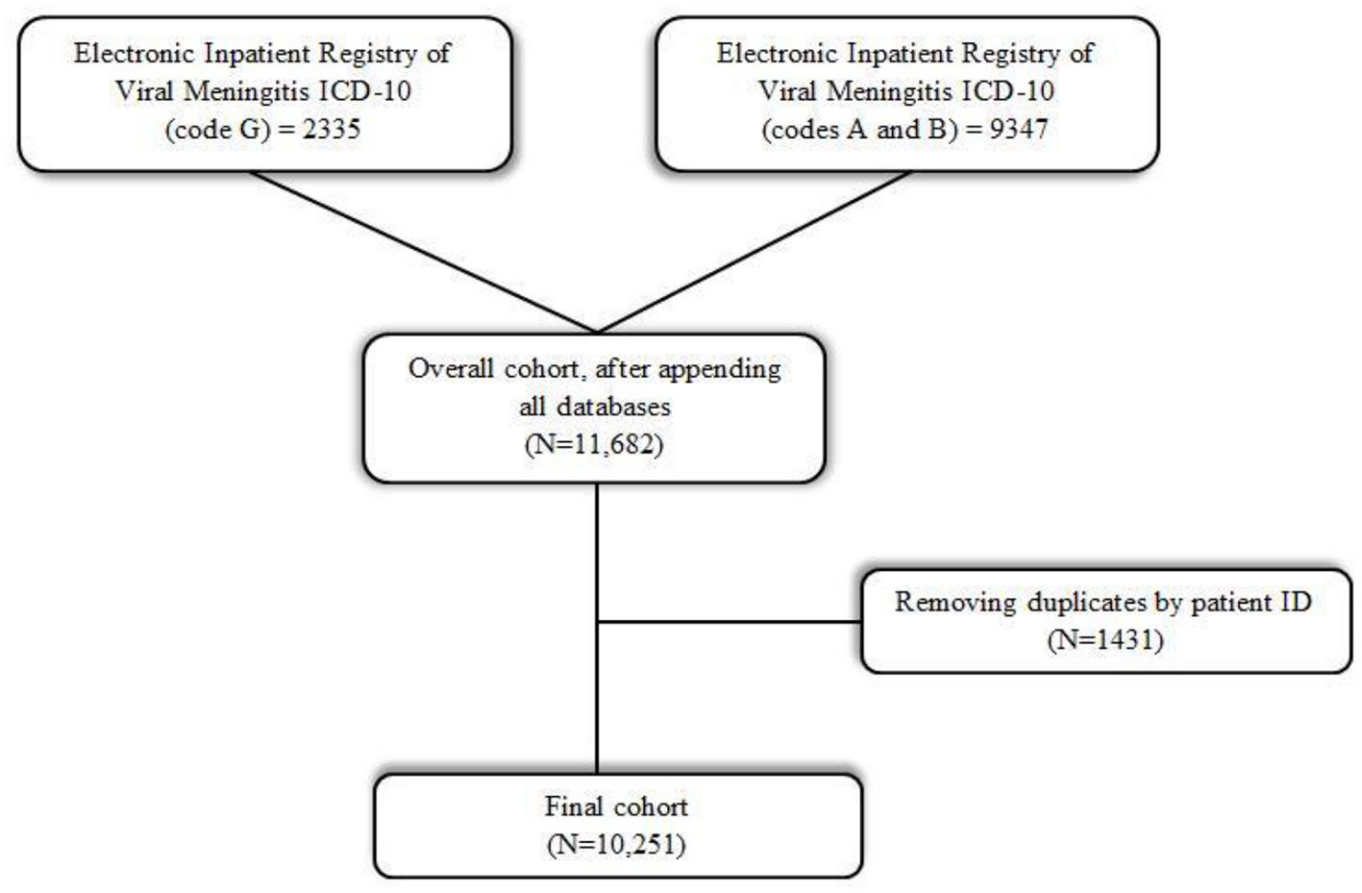
The flowchart diagram.

The raw data of 11,682 patients were retrieved from the Unified National Electronic Health System (UNEHS) and linked with the records to the “Electronic Registry of Inpatients,” which collects general health records from medical institutions. The birth and death statistics were obtained independently from the “Registry of Attached Population” and linked to the hospitalized patients through a Population Registry Number (RPN ID). Duplicate data containing the same population registry numbers (RPN ID) were eliminated. Finally, 10,251 out of 11,682 patients were included for further statistical evaluation (Figure 1). Kazakhstani population characteristics were gathered from the Statistics Committee (14). Meteorological data, including average temperature (°C), average atmospheric pressure (hPa), and average relative humidity (%) were taken from the “Reliable Prognosis” website (15). The study was approved by the Institutional Review and Ethics Committee of the Nazarbayev University (NU-IREC 315/21092020 on 23/09/2020) with exemption from informed consent, as the study involved secondary data that was derived from the UNEHS. Patients were not involved in the study.

### 2.2. Exposures and covariates

The registry included data on the dates of admission and discharge, outcomes of discharge, RPN ID, ICD-10 codes, and some demographic parameters. Demographic data included age, sex, ethnicity, residency setting, and regions. They were categorized by age [children (<1–9 y.o.), adolescents (10–17 y.o.), adults (18–59 y.o.), and elderly (>60 y.o.)]; ethnicity (Kazakhs, Russians, and others); residency (rural and urban) and region which was divided as cities of republican significance with a population above one million (Nur-Sultan, Almaty and Shymkent cities), and by geographical location as North Kazakhstan (Kostanay, Akmola, Pavlodar, and North Kazakhstan regions), South Kazakhstan (Kyzylorda, Turkestan, Zhambyl and Almaty regions), Central Kazakhstan (Karaganda region), East Kazakhstan (East Kazakhstan region) and West Kazakhstan (Atyrau, Aktobe, Mangystau, and West Kazakhstan regions). For all periods, hospitalization-related variables such as dates of hospitalization, length of stay in days, discharge outcome (out-of-hospital deaths, in-hospital deaths, and alive), and final diagnosis were collected.

### 2.3. Outcome assessment

The incidence and in-hospital mortality of VM patients were assessed. The incidence rate was calculated by dividing the number of newly-diagnosed patients by the annual average total general population size. The mortality rate was computed using the assumption that in-hospital mortality equals the number of deaths caused by a particular disease relative to the number of cases of a given disease in a defined period of time (16), hereinafter referred to as the case-fatality rate, which was obtained by dividing the number of deaths by the number of newly-diagnosed cases.

The beginning of the follow-up period was the date of the first admission to the hospital, and patients' data followed until death, or the end of the follow-up period (December 31, 2019). For the outcome variable, in-hospital mortality (deaths during hospitalization) was of interest and the date of hospital discharge was used for censoring the in-hospital mortality survival analysis.

### 2.4. Statistical analysis

Data were reported as absolute values and percentages for categorical variables. Incidence and in-hospital mortality rates within the VM population are presented as per 100,000 population. Continuous variables were described using means and standard deviations, whereas skewed continuous variables were represented using medians and interquartile ranges (IQR). Analysis of variance (ANOVA) was used to examine the relationship between variables such as duration and outcome of stay (number of in-hospital and out-of-hospital deaths). Bonferroni's correction for multiple comparisons was used for age groups.

The association between climatic parameters and VM was then examined using Spearman's correlation coefficient matrix. Considering that the incidence data of infectious diseases are often over-dispersed and the assumption of Poisson regression is violated, we used a negative binomial regression to investigate the independent influence of fluctuations in daily meteorological parameters on VM morbidity, where the response variable of interest is number of cases and the covariates are daily average temperature, air pressure and humidity with lags. Given that the majority of infected persons frequently have an incubation period spanning from 3 to 10 days, we included climatic parameters with an average lag of 7 days in our regression model. Based on the regression coefficients, the incident rate ratio (IRR) and its related 95 % confidence intervals (95% CI) were calculated to determine the independent contribution of climatic conditions to the incidence of VM.

The Kaplan-Meier estimation and Log-rank test were used to demonstrate the crude survival and statistically significant differences. After confirming its assumptions, Cox proportional hazards regression analysis was utilized to calculate crude and adjusted hazard ratios. The full model was adjusted for sex, age categories, ethnicity, residency, regions, and admission type. For all analyses, *P-*values are two-sided and presented as statistically significant at <0.05. The corresponding maps were constructed using the QGIS 3.14.1-Pi version. All statistical analyses and data management were carried out using STATA 16.1 MP2 Version (STATA Corporation, College Station, TX).

## 3. Results

### 3.1. Comparison of demographic and disease-related characteristics for hospital admissions by age groups

The socio-demographic characteristics of the cohort (*n* = 10,251) are given in Table 1. During 2014–2019, there were 5,264 (51.35%) children, 2,708 (26.42%) adolescents, 2,166 (21.13%) adults and 113 (1.1%) elderly people diagnosed with VM. The youngest and oldest patients have a mean age of 5 and 67, respectively. There were 4,321 (42.15%) females and 5,930 (57.85%) males in the cohort. 71.03% of patients were ethnic Kazakhs, 13.44% were of Russian ethnicity and 8.43% were listed as other ethnicities. Overall, 8,806 (85.9%) patients of the cohort are from urban residencies.

**Table 1:** Socio-demographic and disease-related variables for age categories of VM (*n* = 10,251).

**Variables no. (%)**	**All cases** ***n*** **= 10,251 (100)**	**Children <1–9** ***n*** **= 5,264 (51.35)**	**Adolescents 10–17** ***n*** **= 2,708 (26.42)**	**Adults 18–59** ***n*** **= 2,166 (21.13)**	**Elderly >60** ***n*** **= 113 (1.1)**
**Age, mean (SD**±**)**	12.8 (11.7)	5.4 (2.4)	12.5 (2.1)	28.5 (9.6)	67.4 (6.5)
**Sex no. (%)**					
Female	4,321 (42.15)	2,088 (39.67)	1,103 (40.73)	1,070 (49.4)	60 (53.1)
Male	5,930 (57.85)	3,176 (60.33)	1,605 (59.27)	1,096 (50.6)	53 (46.9)
**Ethnicity no. (%)**					
Kazakh	7,281 (71.03)	3,706 (70.4)	1,972 (72.82)	1,553 (71.7)	50 (44.25)
Russian	1,378 (13.44)	623 (11.84)	377 (13.92)	338 (15.6)	40 (35.4)
Other	864 (8.43)	352 (6.69)	226 (8.35)	263 (12.14)	23 (20.35)
Not reported	728 (7.1)	583 (11.08)	133 (4.91)	12 (0.55)	0
**Resident no. (%)**					
Urban	8,806 (85.9)	4,686 (88.02)	2,265 (83.64)	1,766 (81.53)	89 (78.76)
Rural	1,445 (14.1)	578 (10.98)	443 (16.36)	400 (18.47)	24 (21.24)
**Number of hospital days, median (IQR)**	10 (8–12)	10 (8–11)	10 (8–11)	11 (9–15)	12 (7–18)
**Outcome at discharge no. (%)**					
Out-of-hospital deaths	132 (1.29)	24 (0.46)	6 (0.23)	79 (3.65)	23 (20.17)
In-hospital deaths	119 (1.16)	37 (0.7)	5 (0.18)	49 (2.26)	28 (24.56)
Alive	10,000 (97.55)	5,203 (98.84)	2,697 (99.59)	2,038 (94.09)	62 (54.87)
**Admission year no. (%)**					
2014	2,221 (21.67)	1,037 (19.7)	655 (24.19)	514 (23.73)	15 (13.27)
2015	1,182 (11.53)	628 (11.93)	252 (9.31)	278 (12.83)	24 (21.24)
2016	903 (8.81)	463 (8.8)	187 (6.91)	230 (10.62)	23 (20.35)
2017	1,271 (12.4)	694 (13.18)	276 (10.19)	278 (12.83)	23 (20.35)
2018	1,377 (13.43)	759 (14.42)	350 (12.92)	254 (11.73)	14 (12.39)
2019	3297 (32.16)	1683 (31.97)	988 (36.48)	612 (28.25)	14 (12.39)
**Region no. (%)**					
Cities of republican significance^*^	6,419 (62.62)	3,765 (71.52)	1,738 (64.18)	889 (41.04)	27 (23.98)
North Kazakhstan	1,112 (10.85)	394 (7.48)	251 (9.27)	452 (20.87)	15 (13.27)
South Kazakhstan	575 (5.61)	266 (5.05)	121 (4.47)	174 (8.03)	14 (12.39)
Central Kazakhstan	1,160 (11.32)	438 (8.32)	364 (13.44)	347 (16.02)	11 (9.73)
East Kazakhstan	608 (5.93)	293 (5.57)	163 (6.02)	135 (6.23)	17 (15.04)
West Kazakhstan	377 (3.68)	108 (2.05)	71 (2.62)	169 (7.8)	29 (25.66)
**Final diagnoses, no. (%)**					
A87.0–Enteroviral meningitis	914 (8.92)	524 (9.95)	268 (9.9)	121 (5.59)	1 (0.88)
A87.2–Lymphocytic choriomeningitis	1 (0.01)	1 (0.02)	0	0	0
A87.8–Other viral meningitis	2,056 (20.06)	851 (16.17)	626 (23.12)	563 (25.29)	16 (14.16)
A87.9–Viral meningitis, unspecified	5,887 (57.43)	3,479 (66.09)	1,585 (58.53)	807 (37.26)	16 (14.16)
B26.1–Mumps meningitis	2 (0.02)	1 (0.02)	1 (0.04)	0	0
G02.0–Meningitis in viral diseases classified elsewhere	25 (0.24)	10 (0.19)	3 (0.11)	9 (0.42)	3 (2.65)
G03.0–Non-pyogenic meningitis	559 (5.45)	140 (2.66)	71 (2.62)	307 (14.17)	41 (36.28)
G03.9–Meningitis, unspecified	807 (7.87)	258 (4.9)	154 (5.69)	359 (16.57)	36 (31.86)

* Nur-Sultan, Almaty, Shymkent.

While comparing the duration of stay and differences between number of in-hospital and out-of-hospital deaths among age groups, results showed a statistically significant difference by mean between groups. Mentioned comparisons possessed a highly significant *p*-value (<0.001). The biggest proportion of patients was registered in 2019 (32.16%) compared to previous periods, particularly in large cities (62.62%) such as Nur-Sultan, Almaty, and Shymkent compared to other areas.

In respect to the final diagnosis, more than half of the patients were diagnosed with *Unspecified VM*, with 66 % being children, followed by *Enteroviral meningitis* (524/914) by the number of cases in the same group.

### 3.2. Circulating VM agents

**Figure 2:**
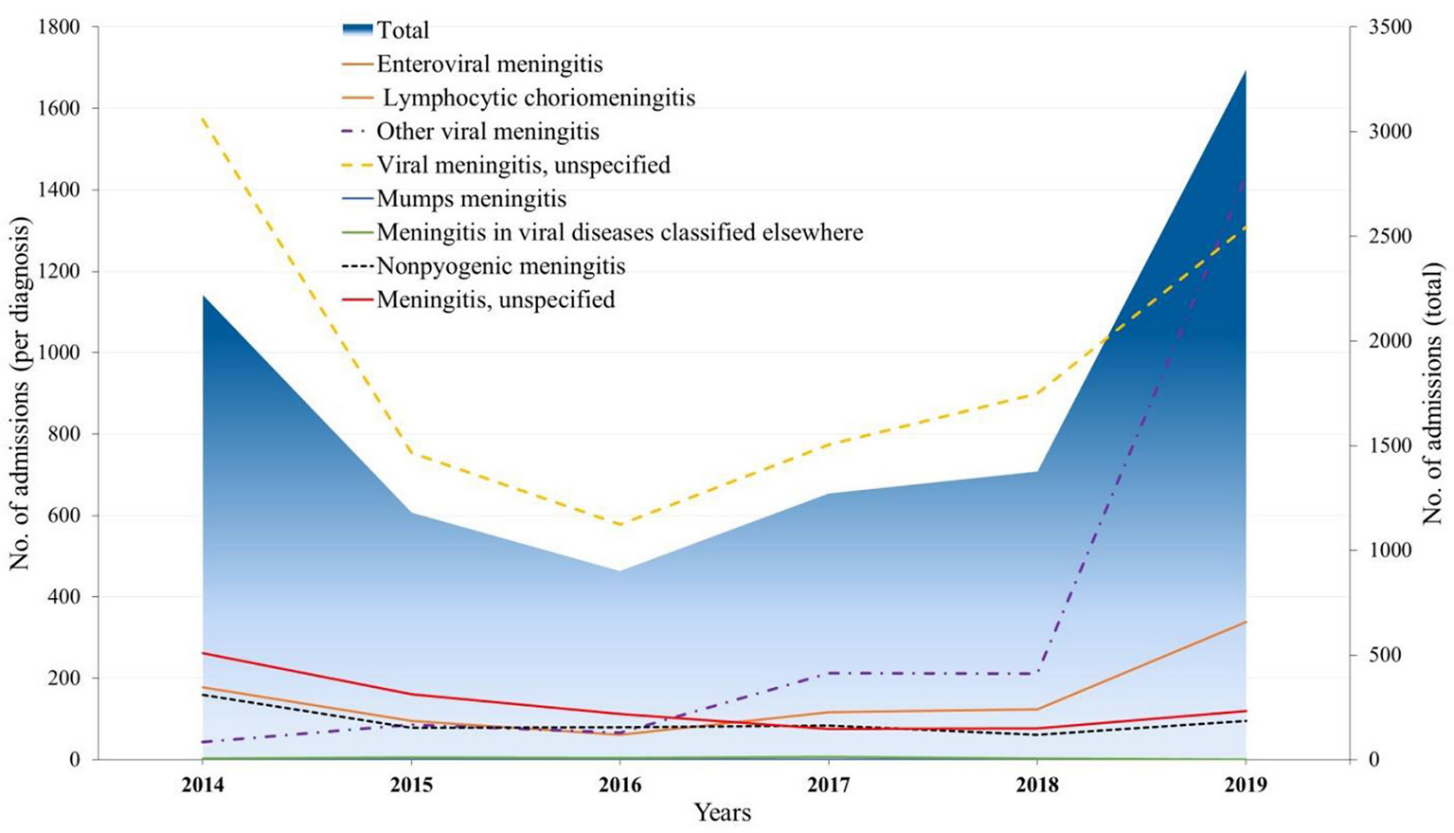
National admission trends with the diagnosis of VM for the period of 2014–2019.

Figure 2 summarizes the distribution of different diagnoses by year of detection. *Unspecified VM* remained the most reported diagnosis until 2019, with a peak registration in 2014 (1,573/2,221, 70.8 %), then *Other VM* diagnosis became prevailing in 2019, followed by *Enteroviral meningitis* in 2014 (178/2,221, 8%), which increased to 10.3 % (338/3,297) in 2019. Furthermore, low admission rates were observed for diagnoses of *Lymphocytic choriomeningitis, Mumps meningitis, Meningitis in viral diseases classified elsewhere*, and *Chronic meningitis* in the period from 2014 to 2019.

### 3.3. Incidence and in-hospital mortality rates

**Figure 3:**
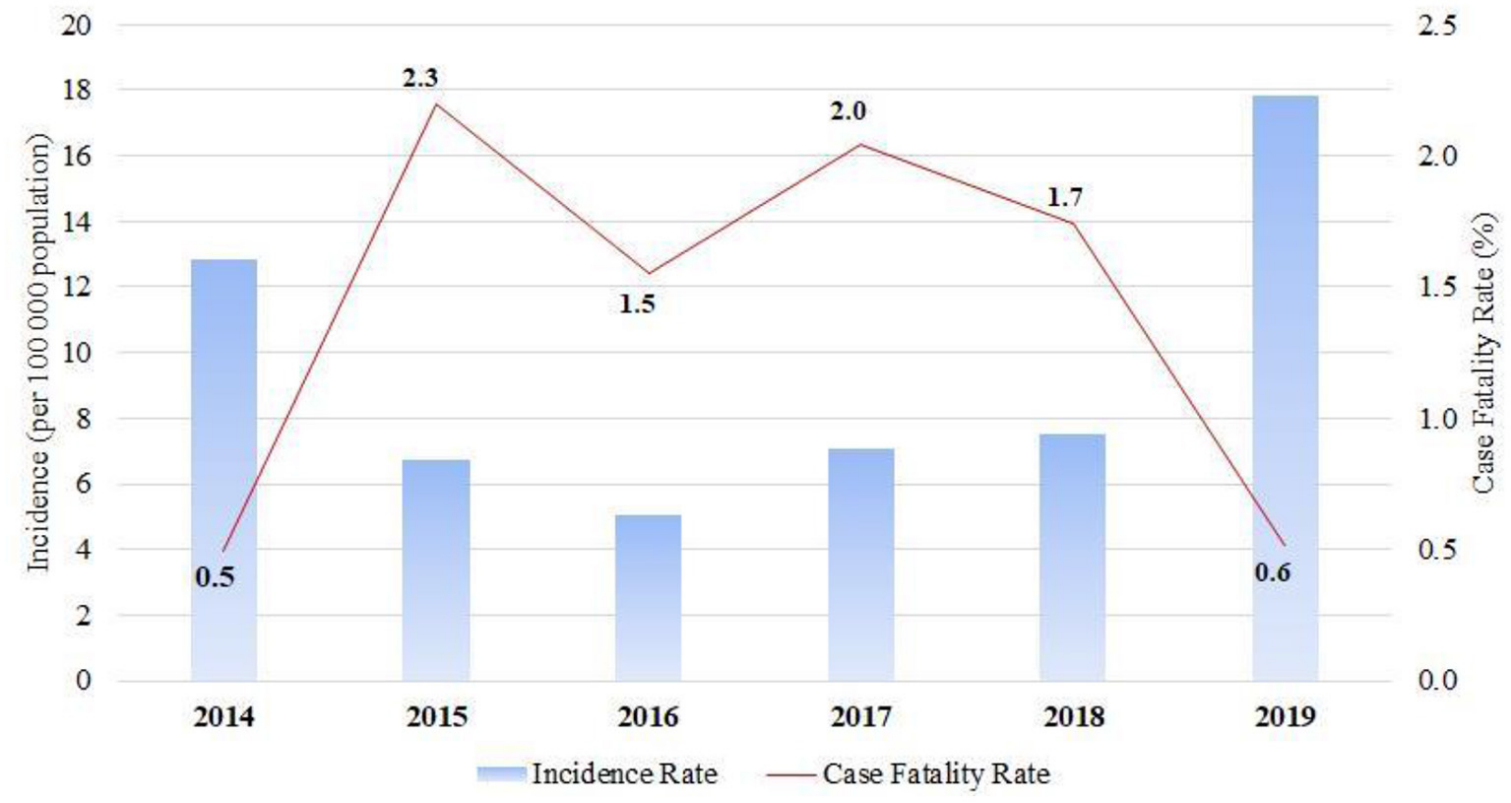
VM Incidence and Case-Fatality Rate over 2014–2019.

**Figure 4:**
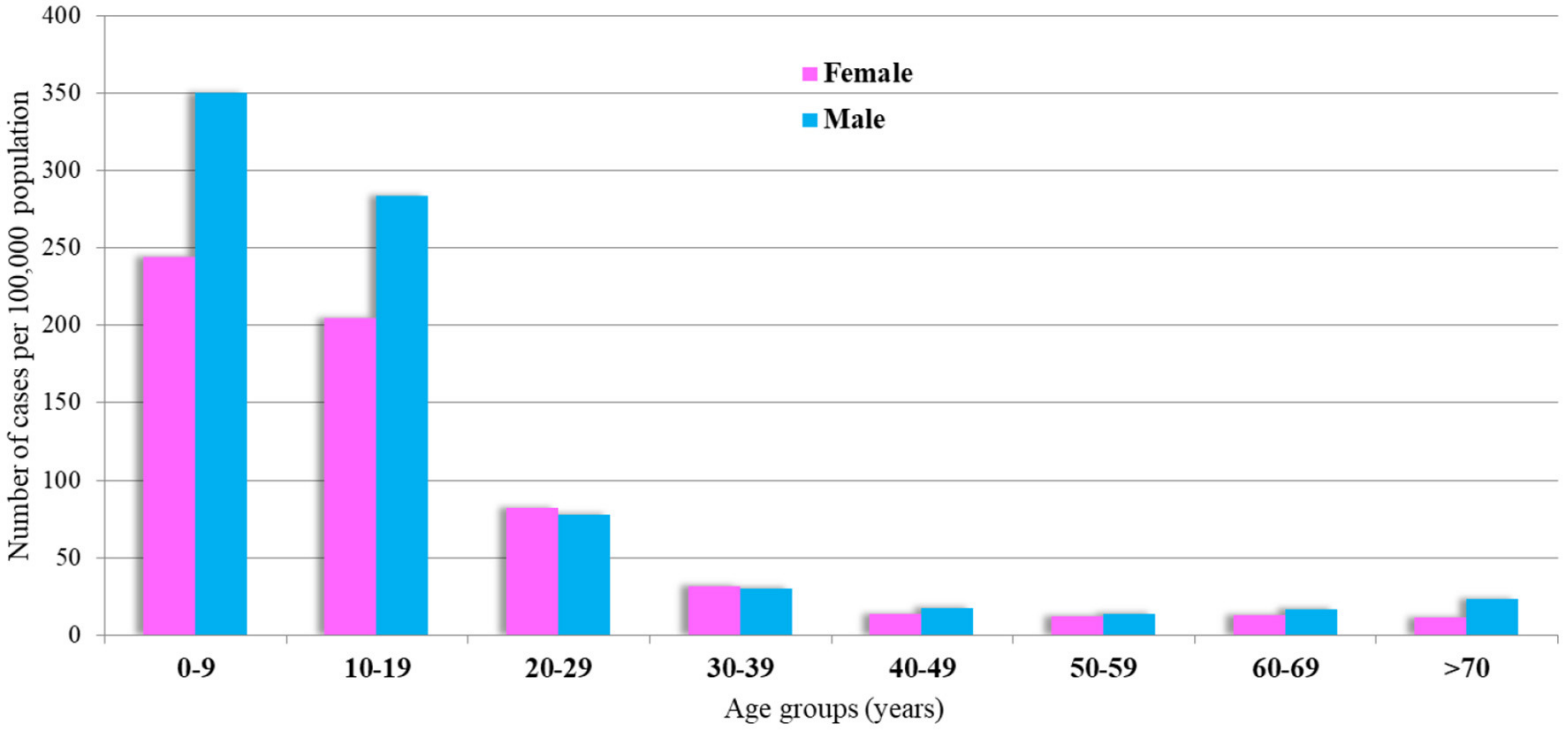
Age and gender-specific incidence rates of VM over 2014–2019.

The incidence rate decreased throughout the first 3 years (2014–2016), reaching a nadir of 5 cases per 100,000 population in 2016, before increasing to 18 cases per 100,000 population at the end of the period (Figure 3). There was a tendency for outbreaks to decline every 5 years, where the highest incidence was 13 per 100,000 population in 2014. The highest age and gender-specific incidence rate (IR) for VM are between 5–and 9 y.o. with 165 and 233 cases per 100,000 population for women and men, respectively (Figure 4). The case-fatality rate changed over time, with the largest adverse outcomes occurring in 2015 and 2017, in 2.3 and 2% of the population, respectively, and with the lowest in 2014 (0.5%) and 2019 (0.6%) (Figure 3).

Incidence and in-hospital mortality by the end of 2019 were higher predominantly in the North (Akmola, Pavlodar, North Kazakhstan region), South (Turkestan, Zhambyl, and Almaty region), Central (Karaganda), East Kazakhstan region, and Nur-Sultan, Almaty and Shymkent cities (Supplementary Figures 1, 2). According to general distributions of VM types by age group and outcome, children were predominantly diagnosed with *VM (unspecified)* and *Other VM*, while the majority of them died from *Meningitis (unspecified), VM (unspecified)*, and *Non-pyogenic meningitis* (Supplementary Figure 3).

### 3.4. Mortality assessment during the period

**Figure 5:**
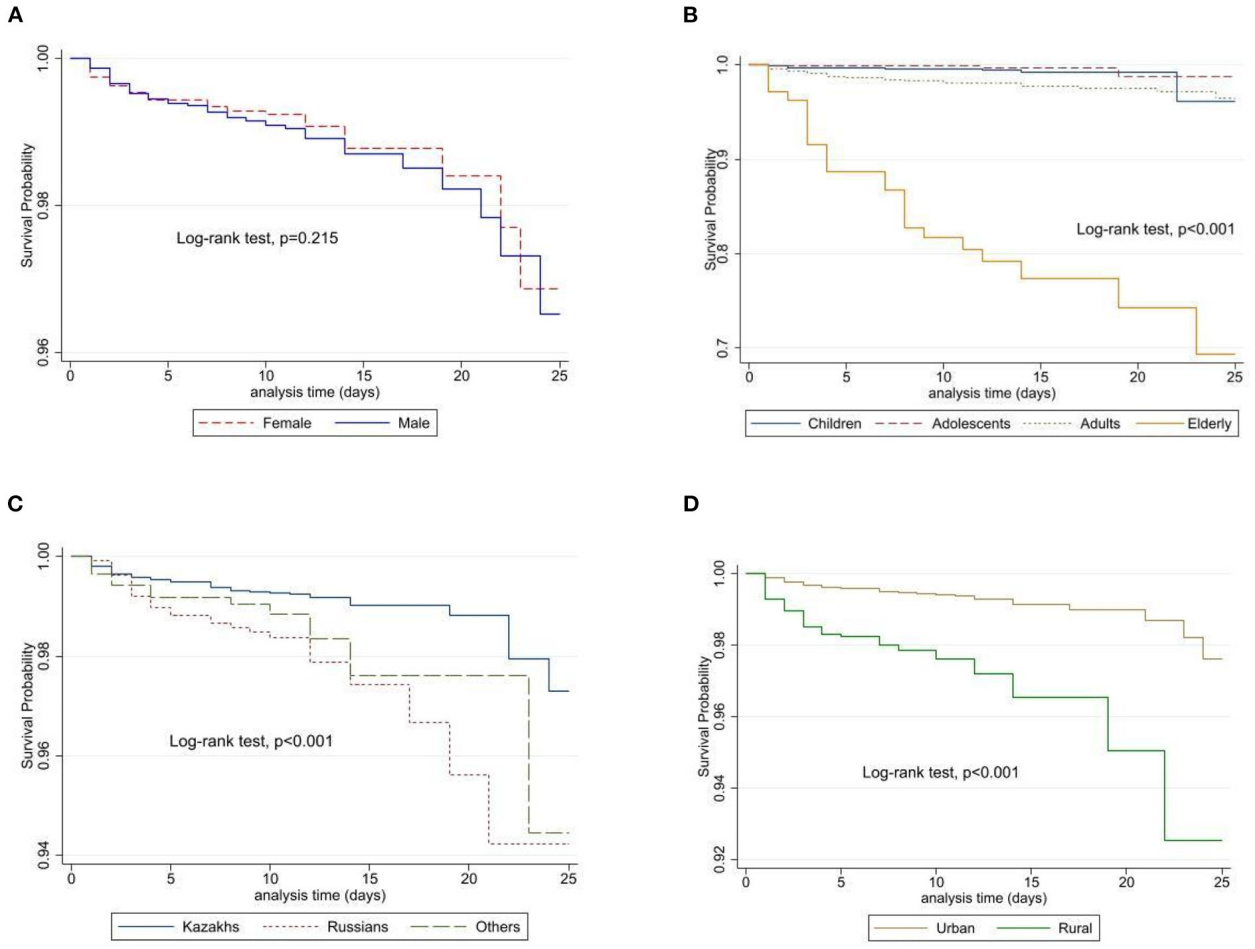
Unadjusted survival probabilities by gender **(A)**, age category **(B)**, ethnicity **(C)**, and place of residence **(D)** among patients in Kazakhstan.

There were 251 deaths reported: 119 cases of in-hospital and 132 cases of out-of-hospital deaths, with a median of 10 [IQR 8–12] days of overall follow-up. Unadjusted Kaplan-Meier analysis (Figure 5) showed that there is no difference in survival probability between males and females (Log-rank test, *p* = 0.215), however, adjusted analysis for age and sex demonstrated that males have a higher risk of death from viral meningitis compared to females.

In both unadjusted and adjusted (for age and sex) regression analysis (Table 2), adolescents, adults, and elderly patients had a 0.3-fold, 3-fold, and <30-fold risk of in-hospital death compared to children (reference). However, in multivariate Cox regression analysis, these results are much lower for adolescents, adults, and elderly patients, 0.3-fold, 2-fold, and 14-fold, respectively. Males had a 35% higher risk of in-hospital mortality compared to females and patients residing in rural areas (compared to city residents) had a 2-fold higher risk of in-hospital mortality. Russians and others [HR = 2.46, (95%CI: 1.5–4.04), *p* < 0.001 and HR = 1.52, (95%CI: 0.83–2.79), *p* < 0.001, respectively] have a higher risk compared to Kazakhs. Living in South and West Kazakhstan was associated with the highest risk with an 8.5-fold and 5.2-fold increase in in-hospital mortality compared to those who were in other regions of Kazakhstan.

**Table 2:** Multivariable Cox proportional hazards regression analysis with unadjusted and adjusted values over 2014–2019.

**Variables no. (%)**	**Unadjusted** **(95% CI)**	***p*** **-Value**	Adjusted for model 1^**^ **(95% CI)**	**p-value**	Adjusted for model 2^***^ **(95% CI)**	***p*** **-Value**
**Age group**		< 0.001		< 0.001		< 0.001
Children	*Ref*.		*Ref*.		*Ref*.	
Adolescents	0.34 [0.13–0.87]		0.33 [0.13–0.87]		0.29 [0.11–0.76]	
Adults	3.16 [1.98–5.04]		3.25 [2.03–5.2]		1.99 [1.24–3.21]	
Elderly	31.62 [18.20–54.91]		33.93 [19.49–59.06]		13.61 [7.65–24.24]	
**Sex**						
Female	Ref.		Ref.		Ref.	
Male	1.28 [0.86–1.89]	0.217	1.56 [1.05–2.32]	0.027	1.35 [0.91–2.01]	0.137
**Ethnicity**		< 0.001		0.134		< 0.001
Kazakh	Ref.		Ref.		Ref.	
Russian	2.24 [1.45–3.47]		1.59 [1.01–2.51]		2.46 [1.5–4.04]	
Other	1.64 [0.91–2.98]		1.13 [0.62–2.08]		1.52 [0.83–2.79]	
**Resident**						
Urban	Ref.		Ref.		Ref.	
Rural	3.55 [2.40–5.24]	< 0.001	3.22 [2.17–4.76]	< 0.001	2.01 [1.29–3.11]	< 0.001
**Region**		< 0.001		< 0.001		< 0.001
Cities of republican significance^*^	Ref.		Ref.		Ref.	
North KZ	5.48 [2.87–10.45]		3.85 [1.99–7.45]		2.62 [1.32–5.18]	
South KZ	14.93 [8.37–26.62]		10.71 [5.95–19.29]		8.54 [4.53–16.13]	
Central KZ	1.33 [0.49–3.57]		1.04 [0.38–2.8]		0.84 [0.31–2.27]	0.726
East KZ	6.7 [3.42–13.12]		5.21 [2.64–10.25]		3.92 [1.94–7.93]	
West KZ	12.69 [6.68–24.13]		6 [3.08–11.68]		5.19 [2.61–10.34]	
**Admission**						
Urgent	Ref.		Ref.		Ref.	
Elective	0.42 [0.056–3.21]	0.406	0.23 [0.03–1.7]	0.15	0.2 [0.03–1.55]	0.125

* Nur-Sultan, Almaty, Shymkent.

** Model 1: adjusted for sex and age category.

*** Model 2: Model 1 + ethnicity, place of residence, region, and admission.

### 3.5. Patterns of the seasonality and correlation analysis of hospitalized patients

**Figure 6:**
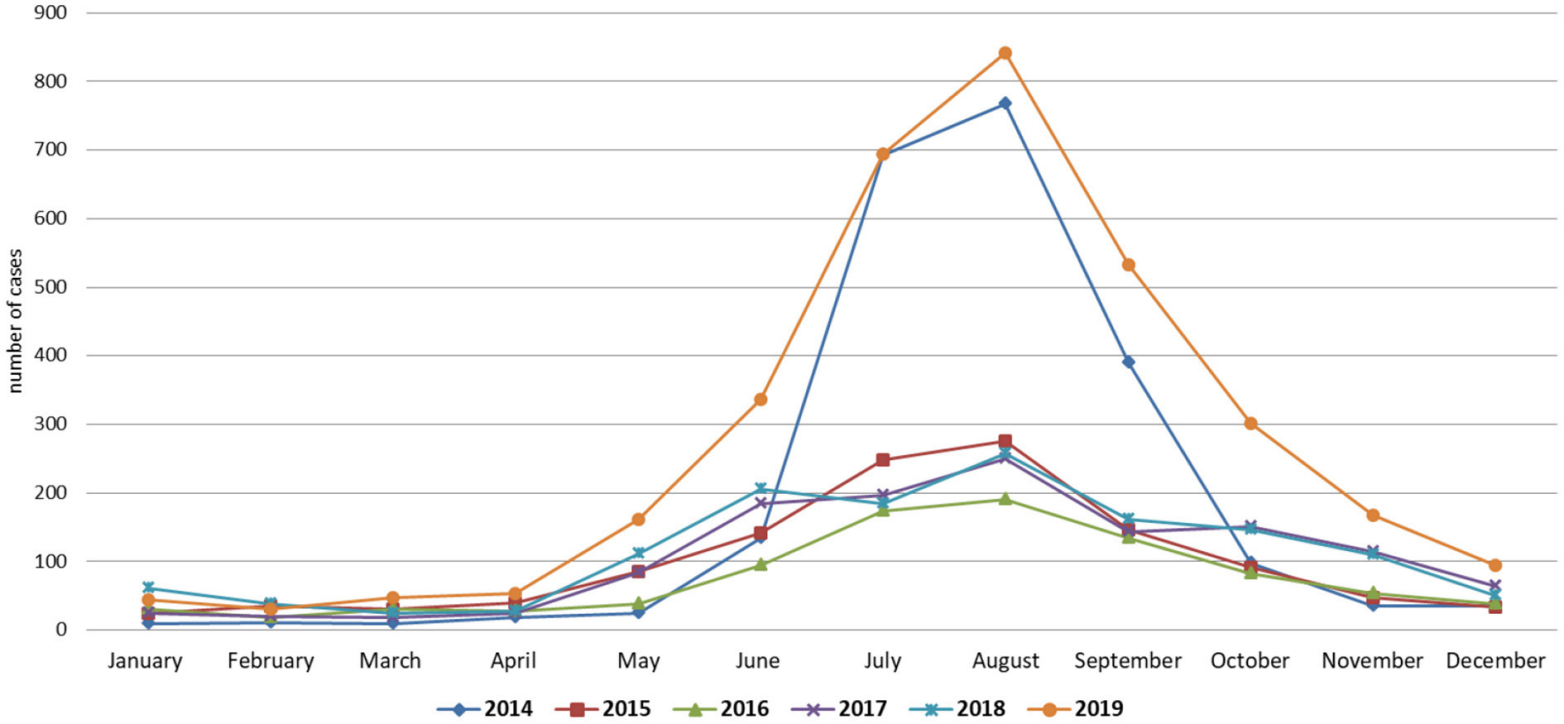
Seasonal dynamics of VM over 2014–2019.

The average atmospheric conditions of the country had been calculated (Table 3). The overall dynamics of VM for 2014–2019 are shown in Figure 6. Data from the 6 years identified significant peaks during the summer seasons (June–August). Starting from April till December the highest numbers of admissions were in the years 2014 and 2019 with the same trend among children and adults (Supplementary Figure 4). When the data were categorized by season (summer: June, July, and August; fall: September, October, and November; winter: December, January, and February; spring: March, April, and May), a close look into 2014 showed the higher proportion of cases in Nur-Sultan, Pavlodar and Karaganda cities between the summer and the end of the fall season (Supplementary Figure 5). Whereas, in 2019 the highest number of cases were registered in Shymkent, Nur-Sultan, and Almaty cities (Supplementary Figure 6). In Almaty, cases were registered throughout the year, whereas in Shymkent and Nur-Sultan cities peak registration came during the summer period.

**Table 3:** Summary statistics for the VM cases and weather parameters in Kazakhstan.

**Variable**	**Mean**	**S.D**	**Min**	**P25**	**P50**	**P75**	**Max**
**Average temperature, (°C)**	16.12	10.11	−32.23	12.2	18.58	23.27	31.01
**Average air pressure, (hPa)**	726.85	11.94	695.2	718.2	725.32	734.99	782.94
**Average relative humidity, (%)**	56.48	15.4	16	44.29	53.69	67.52	100

SD, standard deviation.

P25, P50 and P75–percentiles.

The climatic parameters indicated above have a moderate interaction with the VM cases. In contrast, there was a negative correlation between the VM cases and the average relative humidity. These findings were consistent with Spearman's correlation coefficient matrix results (Table 4).

**Table 4:** Spearman's correlation coefficient matrix between variables and the collinearity statistics.

	**VM cases**	**Temperature (°C)**	**Air pressure (hPa)**	**Humidity (%)**	**VIF**
**VM cases**	1				
**Average temperature, (°C)**	0.3646	1			2.87
**Average air pressure, (hPa)**	0.0197	−0.3205	1		1.18
**Average relative humidity, (%)**	−0.307	−0.7987	0.3024	1	2.7
**Tmean, 3-day lag**	0.3693	0.9629	−0.3206	−0.7526	-
**Tmean, 4-day lag**	0.3699	0.9527	−0.3206	−0.7396	-
**Tmean, 5-day lag**	0.3704	0.9433	−0.3193	−0.7274	-
**Tmean, 6-day lag**	0.3713	0.9351	−0.3186	−0.7164	-
**Tmean, 7-day lag**	0.3729	0.9283	−0.3189	−0.7075	-

VM, Viral Meningitis; VIF, variance inflation factor, p < 0.001.

**Figure 7:**
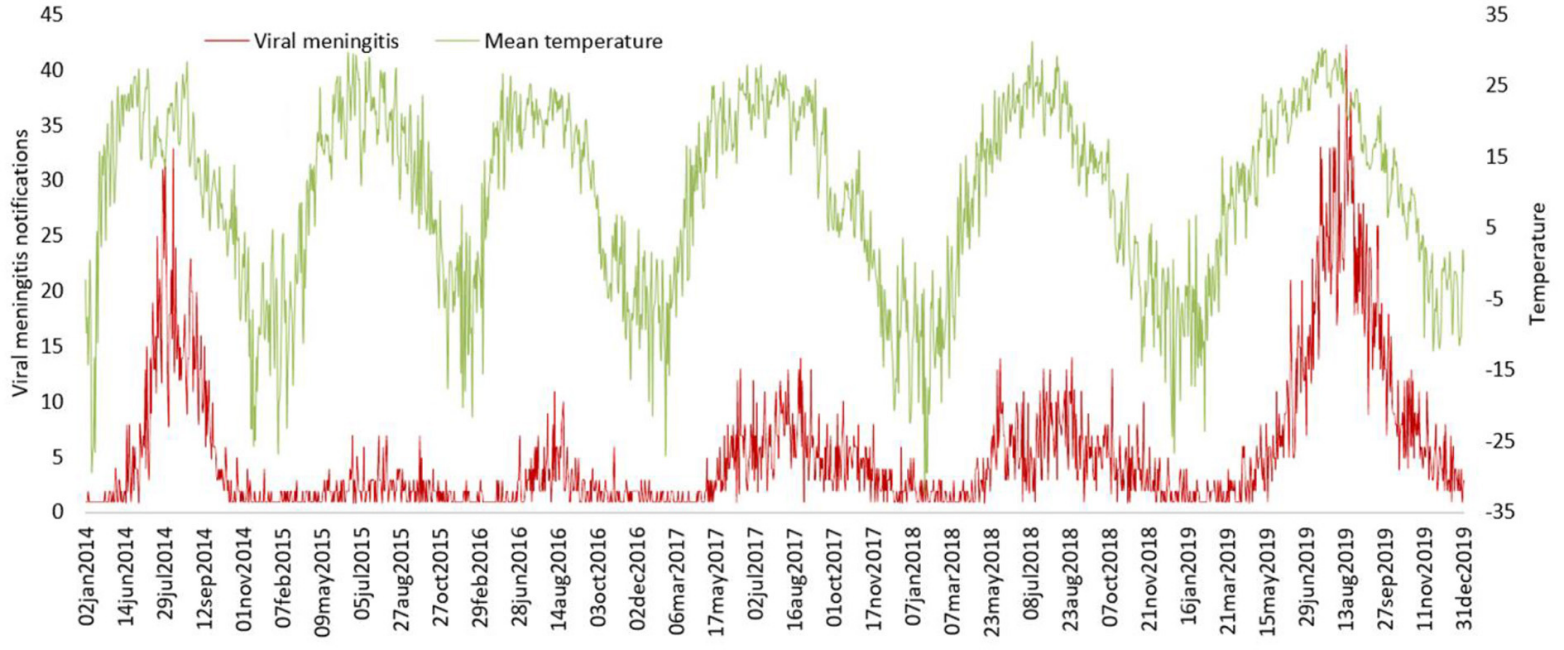
The trends of viral meningitis notifications and temperature metrics between 2014 and 2019.

Negative binomial regression results shows that 1°C increment in the daily average temperature might be associated with an increase in the daily rate of VM cases with IRRs of 1.05 (95% CI 1.047–1.051), 1 hPa increment in the average air pressure and 1% increment in the daily average humidity might contribute to a decrease in the daily rate of VM cases with IRRs of 0.997 (95% CI 0.995–0.998) and 0.982 (95% CI 0.981–0.983), respectively. Analyses of seasonal variations for viral meningitis and temperature illustrated clear seasonal patterns over the study period (Figure 7).

## 4. Discussion

Over the 6-year study period, there was a downward trend in incidence with the lowest rate of 5 cases per 100,000 population in 2016 and the highest incidences of 13 and 18 cases per 100,000 population in 2014 and 2019, respectively. In 2014, the majority of cases were recorded in the cities of Nur-Sultan, Pavlodar, Karaganda, and in 2019–Shymkent, Nur-Sultan, and Almaty. We observed a 35% higher risk of in-hospital mortality in males compared to females. Russians and other ethnicities had a 2.46-fold and 1.52-fold higher risk of in-hospital mortality compared to the Kazakhs. Patients in rural locations and older patients had a 2-fold and 14-fold higher risk of in-hospital mortality than city residents and younger patients, respectively.

Our study documented greater incidence of VM among males (57.85% of all cases) which is consistent with previous observations from MENA (the Middle East and North Africa) studies, such as Oman (2000–2005) (17), Tehran (1999–2005) (18), Lebanon (2008–2016) (19), and Saudi Arabia (1994–1996) (20). Males were also found to be predominantly affected by the viral meningitis epidemic in Romania (21). However, in Michigan, both males and females were diagnosed with VM in equal percentages (10).

This study demonstrated higher registration in children with a mean age of five and they were the largest group (51.35%) diagnosed with *Enteroviral meningitis* compared to others. These findings were in line with previous research in the Gulf area between 2000 and 2005 (22) which stated that children and young people are the most impacted age group. Similarly, a Tunisian investigation found that the average age of VM-infected individuals was 6.1 years of age (23). However, in many countries, including the United States (24) the United Kingdom (25) and Denmark (26) as well as countries like Kuwait (27) and Palestine (28) studies indicate that newborns under the age of 1 year are the most afflicted age group due to their underdeveloped immune system. According to Moore (1982), young children are known to be the principal reservoir of human enteroviruses (29). Additionally, young age is thought to be a risk factor for viral meningitis, with various studies indicating that age-specific incidence is higher in infants and school-aged children of 5–10 years of age (30). It is consistent with our findings that the greatest age-specific incidence rate (IR) for VM was found in children aged 5–9.

Our results show that Russians and other ethnicities had a higher risk of in-hospital mortality compared to Kazakhs. It is difficult to isolate the role of race alone in explaining differences in survival since other social determinants such as socioeconomic status, education level, employment status, dietary patterns, and age-specific incidences must be considered.

The South Kazakhstan area was found to be the most affected by the burden of encephalitis and meningitis (EM) in the country (31). This is consistent with our findings, which found the greatest numbers of cases registered in Almaty and Shymkent, both of which are located in the southern part of the country. Such a tendency can be attributed to the highest population density in the region and milder climates with longer summers, which facilitates easy viral spread and hosts longer endemic periods.

Previous research studies (3, 8) revealed that the majority of patients have an unspecified or rather undetermined kind of VM, which challenges its categorization. According to Husbun et al., about one-quarter of meningitis cases lack an identified cause (32). Unspecified, or Other VM consists of the major part of our data. The unspecificity of final diagnoses in our study findings is likely related to the unavailability of virus-specific nucleic acid amplification (NAATs) and PCR tests needed to determine the specific cause of viral meningitis in Kazakhstan. There is a concern for underdiagnosing VM caused by *Varicella zoster virus, Measles, and Mumps* due to reported poor compliance with childhood vaccination in the country (33, 34), and also unavailability of Chickenpox vaccination in the national immunization program. Furthermore, we believe that discrepancies in proportions of various etiological types may be attributable to difficulties in identifying cases of the disease, economic disparities in hospital admission in rural areas, and possibly patient factors such as refusal of lumbar puncture, etc.

We observed a decreasing secular trend, with outbreaks every 5 years, comparable to VM rates reported in numerous MENA regions, including Lebanon (82.7%; 2008–2016) (19) and Oman (31%; 2000–2005) (17). Aside from the MENA region, other Western countries have reported high VM cases, such as the England and Wales research (2011–2014) with 36% positive VM cases (30) and a Canadian study (1998–2007) with 30% positive VM cases (35). Many factors contribute to the secular trend, such as endemic and epidemic circulation of some serotypes, and emergence of “new” serotypes. In addition, advances in public hygiene throughout the research period may have played a role. Because enterovirus primarily spread *via* the fecal-oral pathway, improved public sanitation could have been a plausible explanation for viral meningitis decline over the research period.

The World Health Organization identified dengue, viral encephalitis, diarrheal illness, enteric fever, pneumonia, and meningitis as the diseases most vulnerable to climate change, and expected a significant increase in incidence in tropical nations (36). Our impression of the seasonality of VM is consistent with overall awareness of higher incidence in warm months and in line with previous reports (37, 38). As far as we know, this is the first study in Central Asia that focuses on VM epidemiology and investigates the association between disease and three meteorological factors.

Our analysis has several limitations. The calculations are based only on hospital admission and discharge status; hence there is a possibility of selection bias with cases of mild viral meningitis not admitted and captured in the study. Furthermore, data from potential individuals who might have died before hospitalization may be missed, leading to an underestimation of the incidence and mortality rates. The survival and regression analyses are limited to used variables. We were unable to obtain cause-specific mortality data and there could be potential errors in misclassification. In addition, lack of information on co-infections, absence of clinical characteristics such as severity of the disease, expanded virus identification and serotyping, laboratory confirmation data, treatment regimens, etc. Since the ICD-10 coding was used and there were diagnoses as *Other VM*, we could not give a clear definite explanation for this coding. Nevertheless, despite these constraints, this is the first Central Asian study to use digital healthcare data to illustrate the epidemiology and impact of climate on VM in Kazakhstan. Throughout the 6 years, we aimed to give a comprehensive study of all hospital admissions linked to ICD-10 codes, along with an analysis of in-hospital mortality.

## 5. Conclusion

The current study assessed the incidence, in-hospital mortality rates, and influence of climate variables on VM infection in Kazakhstan from 2014 to 2019. There was a tendency to decrease the incidence with outbreaks every 5 years, and mortality rates were higher for Russians and other ethnicities compared to Kazakhs, for males compared to females, for elder patients compared to younger patients, and for patients living in rural areas compared to city residents. The climatic parameters and the days of delay indicated a moderate interaction with the VM cases. These findings can be considered as a starting point to guide clinicians, public health experts, and policymakers to understand the epidemic patterns of VM better when it comes to planning the prevention and control of infectious illnesses and to conduct a study in the future using a wide range of climatic indicators. Special attention should be paid to laboratory analysis and identification of viral pathogens and serotyping in order to estimate the true incidence of infection in different regions and implement appropriate public health measures.

## Data availability statement

The original contributions presented in the study are included in the article/Supplementary material, further inquiries can be directed to the corresponding author.

## Author contributions

SY, AZ, AI, and AGa: conceptualization and methodology. SY, AGu, YS, and AAl: software. SY, AZ, AI, AGu, YS, GZ, KM, DS, AAl, AS-S, AAb, and AGa: validation. SY, AGu, and YS: formal analysis. SY and AZ: resources and writing—original draft preparation. SY, AGu, YS, GZ, KM, DS, and AAl: data curation. AI, AAb, AS-S, and AGa: writing—review and editing. AGa: supervision, project administration, and funding acquisition. All authors have read and agreed to the published version of the manuscript.
