# Supplementary material for: Epidemiological characteristics and climatic variability of viral meningitis in Kazakhstan, 2014–2019
Source: Front Public Health. 2023 Jan 4;10:1041135. doi: 10.3389/fpubh.2022.1041135 (PMC9845948; doi:10.3389/fpubh.2022.1041135)
Supplement: Supplementary file 1 [file Data_Sheet_1.pdf]

Supplementary Material

1. Supplementary Figures

A.

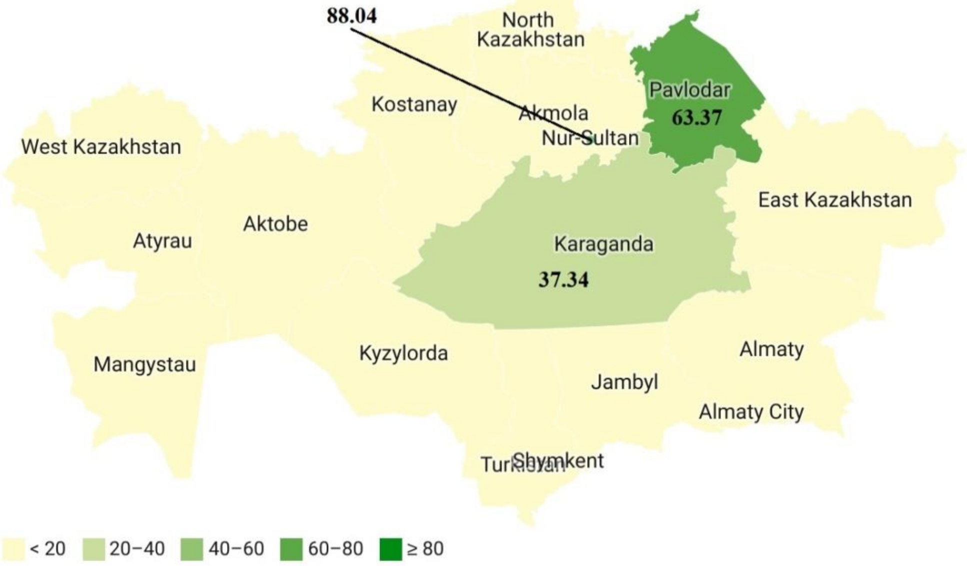

B.

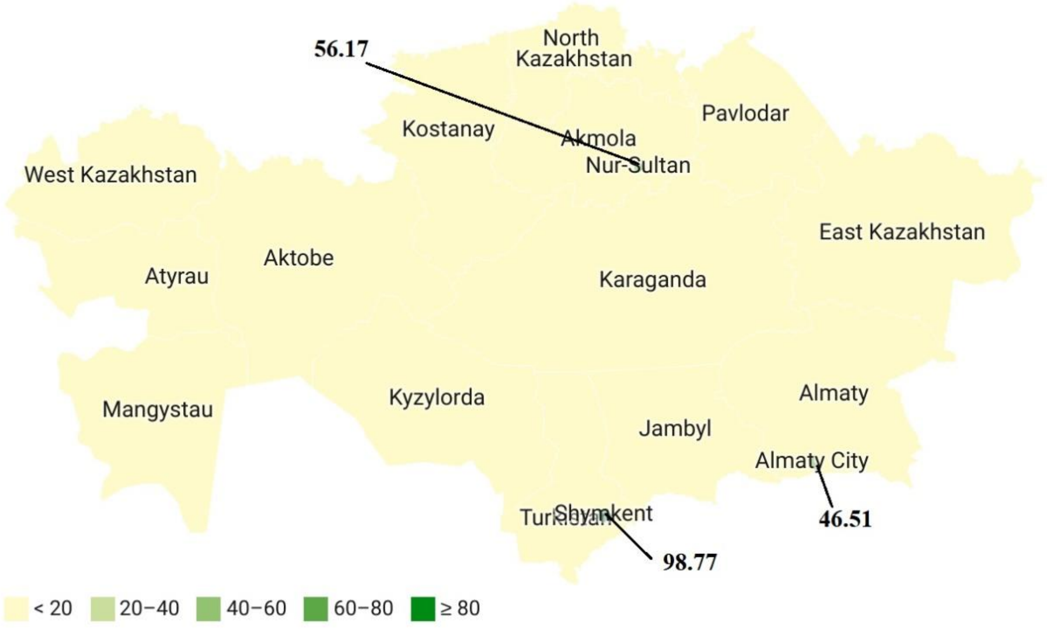

Supplementary Figure 1. The incidence rate of VM in Kazakhstan regions in 2014 **(A)** and 2019 **(B).**

A.

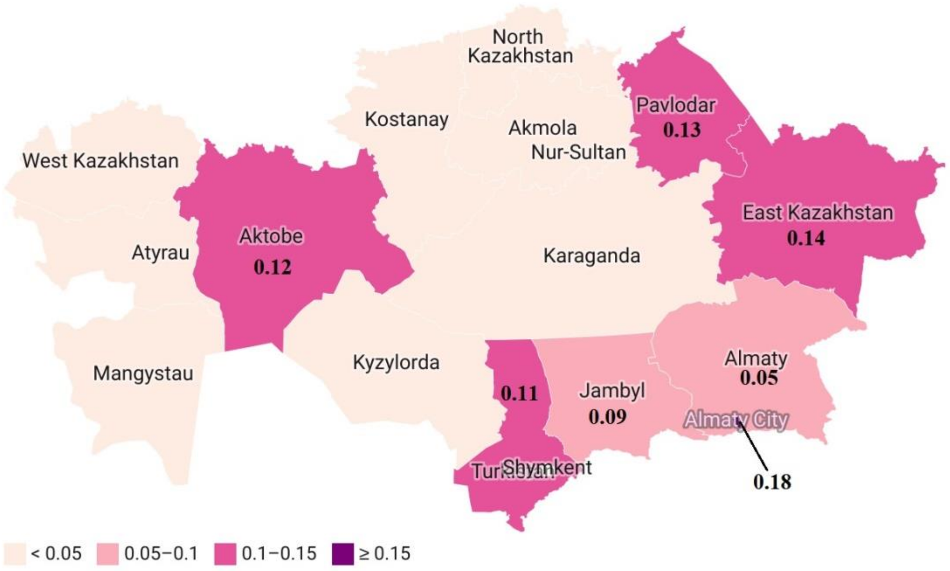

B.

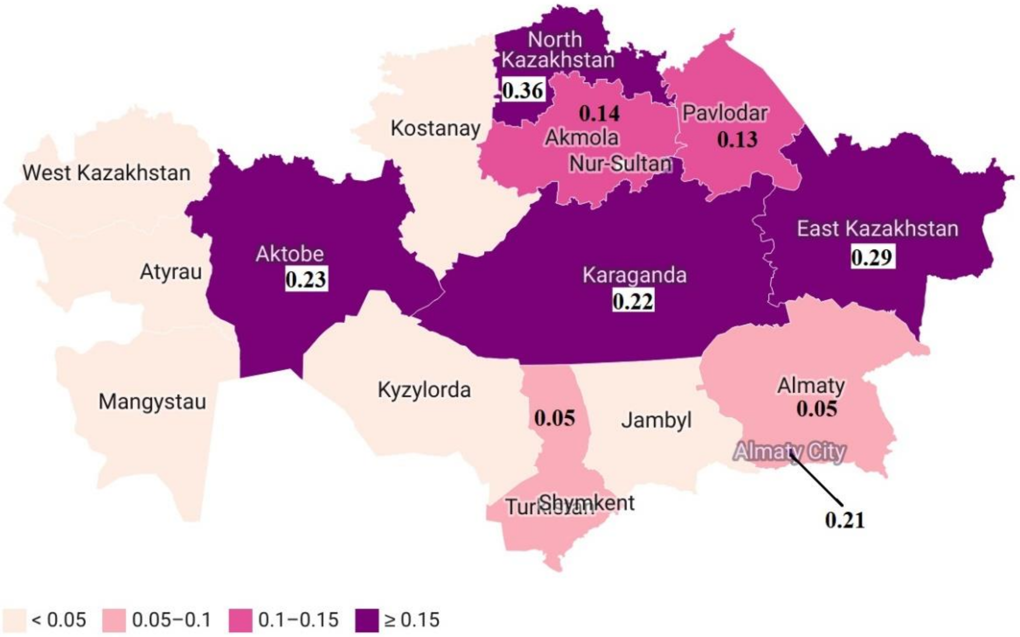

Supplementary Figure 2. In-hospital mortality rates of VM in Kazakhstan regions in 2014 **(A)** and 2019 **(B).**

A.

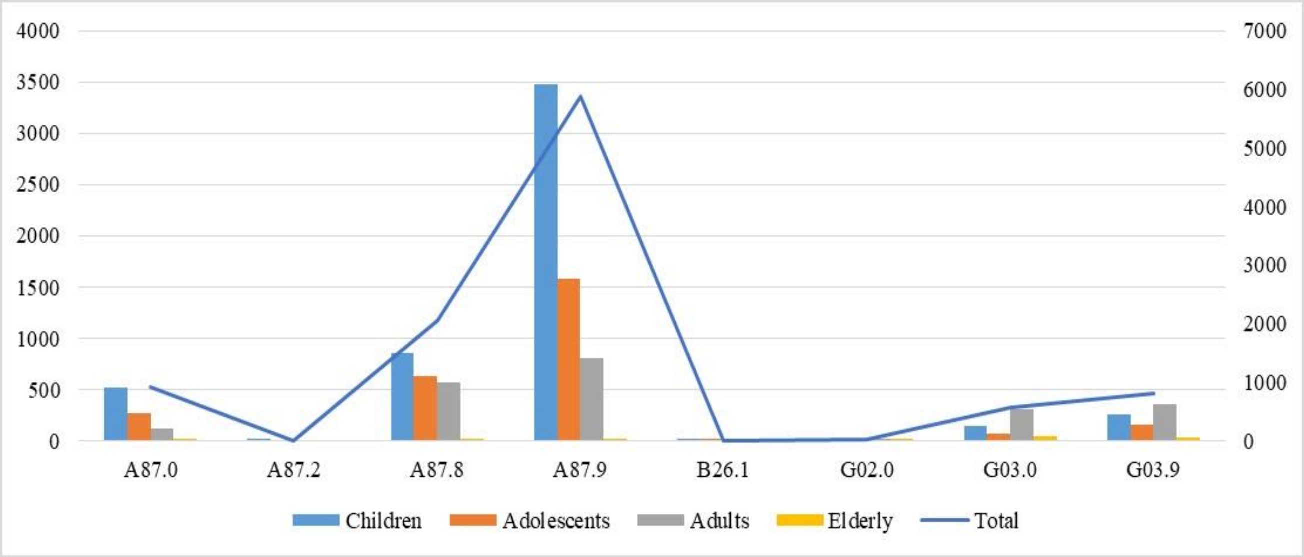

B.

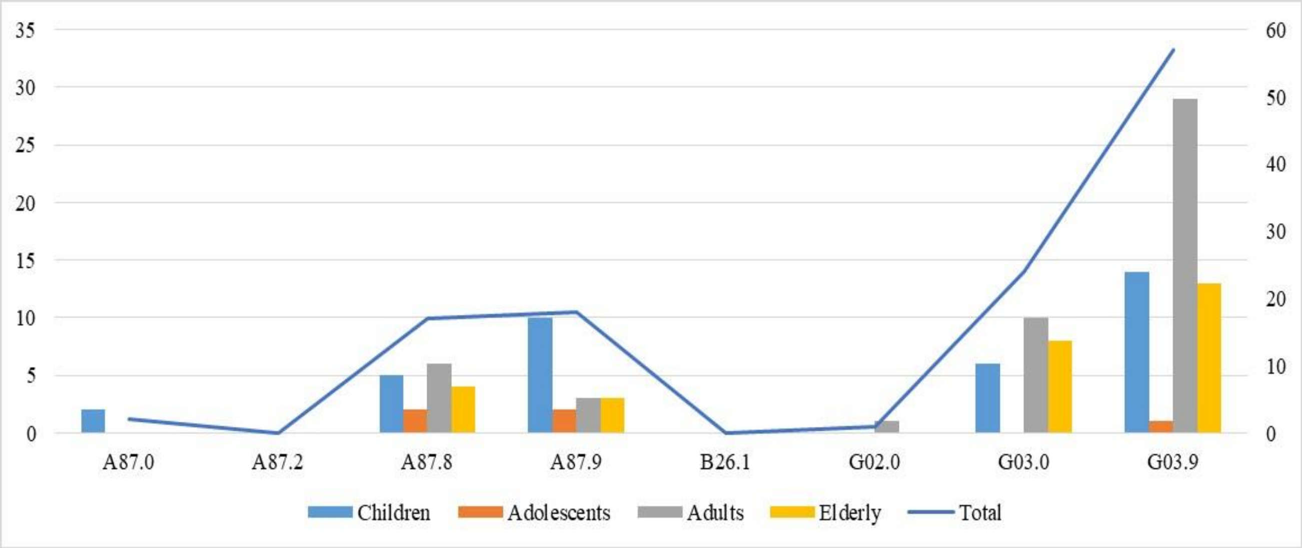

Supplementary Figure 3. The general distributions of VM types by age group **(A)** and outcome (in-hospital death) **(B).** A87.0 - Enteroviral meningitis; A87.2 - Lymphocytic choriomeningitis; A87.8 - Other viral meningitis; A87.9 - Viral meningitis, unspecified; B26.1 - Mumps meningitis; G02.0 - Meningitis in viral diseases classified elsewhere; G03.0 - Nonpyogenic meningitis; G03.9 - Meningitis, unspecified.

A.

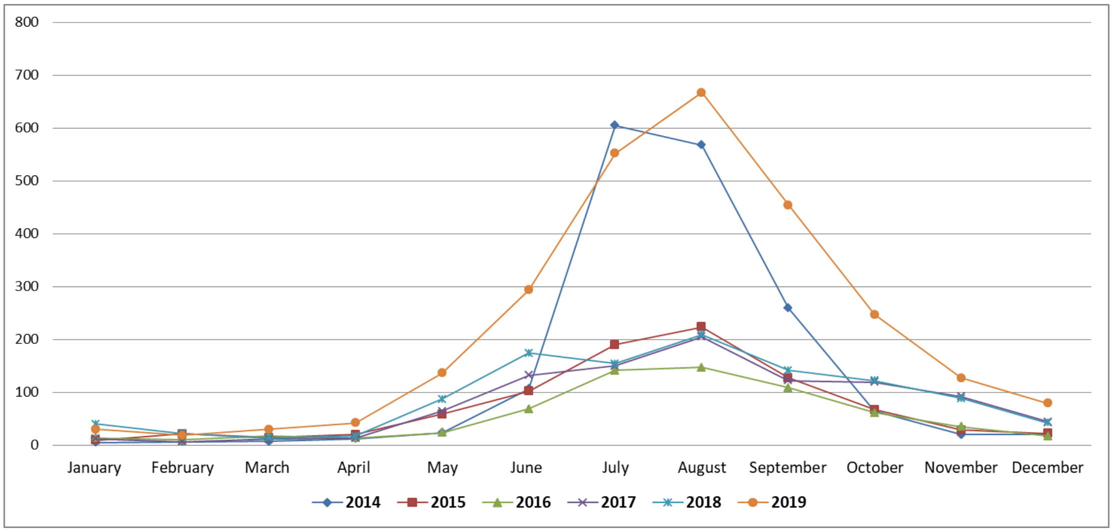

B.

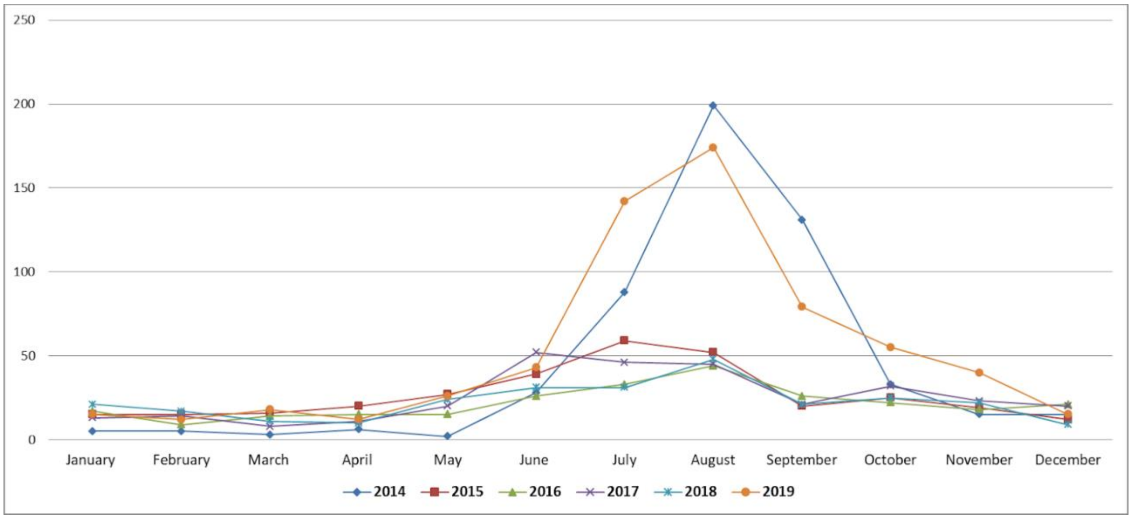

Supplementary Figure 4. Seasonal dynamics of VM in children **(A)** and adults **(B)** between 2014 and 2019.

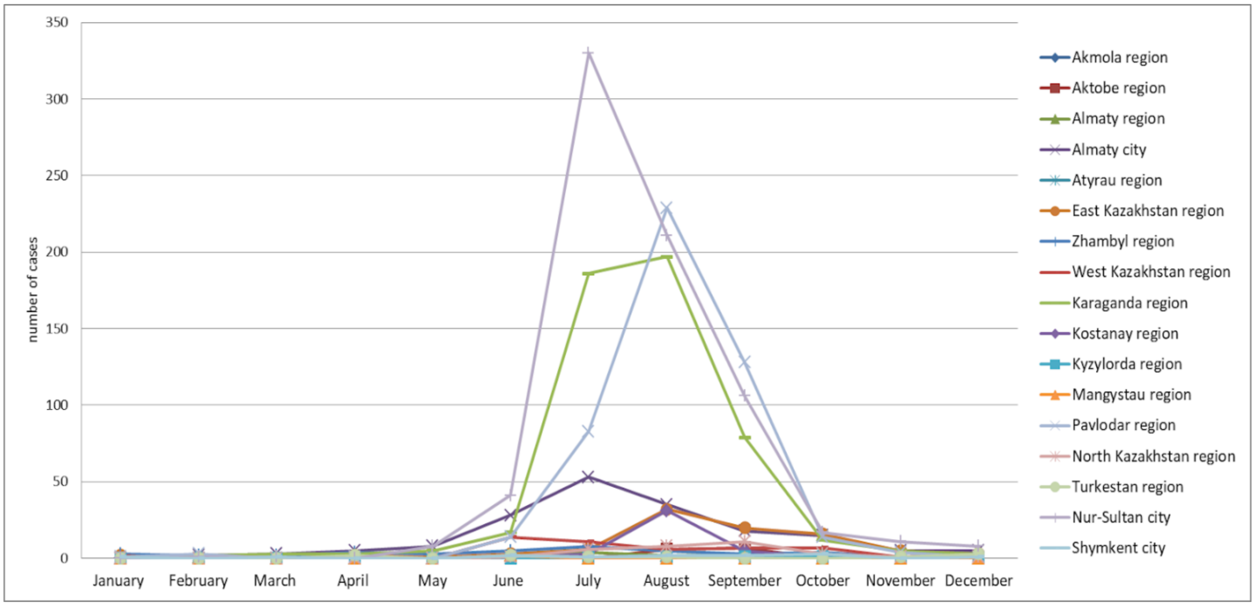

Supplementary Figure 5. Seasonal dynamics of VM in 2014.

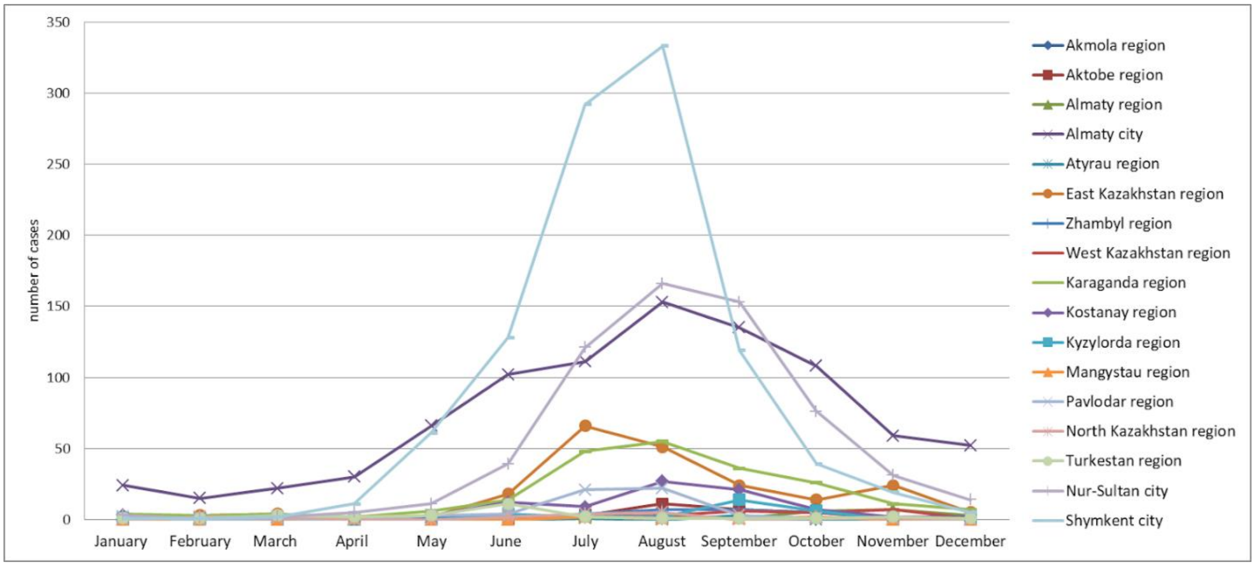

Supplementary Figure 6. Seasonal dynamics of VM in 2019.
